# Machine Learning Identifies a Signature of Nine Exosomal RNAs That Predicts Hepatocellular Carcinoma

**DOI:** 10.3390/cancers15143749

**Published:** 2023-07-24

**Authors:** Josephine Yu Yan Yap, Laura Shih Hui Goh, Ashley Jun Wei Lim, Samuel S. Chong, Lee Jin Lim, Caroline G. Lee

**Affiliations:** 1 Department of Biochemistry, Yong Loo Lin School of Medicine, National University of Singapore, Singapore 117596, Singapore; 2 NUS Graduate School, National University of Singapore, Singapore 119077, Singapore; 3 Department of Paediatrics and Obstetrics & Gynaecology, Yong Loo Lin School of Medicine, National University of Singapore, Singapore 119074, Singapore; 4 Division of Cellular & Molecular Research, Humphrey Oei Institute of Cancer Research, National Cancer Centre Singapore, Singapore 168583, Singapore; 5 Duke-NUS Medical School, Singapore 169857, Singapore

**Keywords:** hepatocellular carcinoma, biomarker, machine learning, exosome, RNA

## Abstract

**Simple Summary:**

Hepatocellular carcinoma (HCC) is the third leading cause of cancer-related death worldwide. HCC is often diagnosed at a late stage when treatment effectiveness is limited and its prognosis remains dire. Exosomes are secreted by all living cells, including cancer cells, and contain biological material with potential to highlight disease conditions or dysregulated pathways involved in tumourigenesis. This study employs artificial intelligence methods to identify a signature of exosomal RNAs from 114,602 exosomal RNAs in 118 HCC patients and 112 healthy individuals that can predict HCC. A signature of nine exosomal RNAs, mainly in the immune, platelet/neutrophil and cytoskeletal pathways, was identified to predict HCC with an accuracy of ~85%. Hence, these nine exosomal RNAs have potential to be developed as clinically useful minimally invasive biomarkers for HCC.

**Abstract:**

Hepatocellular carcinoma (HCC) is the third leading cause of cancer-related death worldwide. Although alpha fetoprotein (AFP) remains a commonly used serological marker of HCC, the sensitivity and specificity of AFP in detecting HCC is often limited. Exosomal RNA has emerged as a promising diagnostic tool for various cancers, but its use in HCC detection has yet to be fully explored. Here, we employed Machine Learning on 114,602 exosomal RNAs to identify a signature that can predict HCC. The exosomal expression data of 118 HCC patients and 112 healthy individuals were stratified split into Training, Validation and Unseen Test datasets. Feature selection was then performed on the initial training dataset using permutation importance, and the predictive performance of the selected features were tested on the validation dataset using Support Vector Machine (SVM) Classifier. A minimum of nine features were identified to be predictive of HCC and these nine features were then evaluated across six different models in an unseen test set. These features, mainly in the immune, platelet/neutrophil and cytoskeletal pathways, exhibited good predictive performance with ROC-AUC from 0.79–0.88 in the unseen test set. Hence, these nine exosomal RNAs have potential to be clinically useful minimally invasive biomarkers for HCC.

## 1. Introduction

Hepatocellular carcinoma (HCC) is the most common primary malignancy of the liver [1]. Despite improvement in treatment options, prognosis remains poor with a high global mortality rate of 9.5 cases per 100,000 patients [2] and a 5-year survival of 18%. This is attributed to a large proportion of patients being only diagnosed at a late stage when there is high likelihood of extra-hepatic metastasis [3]. It is further observed that early diagnosis with curative treatment increases the 5-year survival rate [4]. As such, various medical bodies have recommended screening for high-risk patients such as those with liver cirrhosis.

The difficulty of implementing screening programmes is in part due to the lack of appropriate screening methods. Clinically, abdominal ultrasound and serum alpha-fetoprotein (AFP) constitute the backbone for HCC screening. However, the sensitivity and specificity of AFP are limited [5] as raised levels of serum AFP are also indicative of chronic infection or deterioration of the liver, with or without the development of HCC. Moreover, some HCC patients retain normal AFP levels throughout progression of disease [6,7,8,9]. As such, the discovery of novel markers that address the low sensitivity and specificity is needed for more accurate screening and diagnosis of HCC.

Exosomes have emerged as a promising source of biomarkers for various cancers, with ongoing clinical trials investigating their use in diagnostics and therapeutics [10,11]. Exosomes are membrane-bound micro-vesicles that range from 40–150 nm in diameter secreted by all living cells and are present in biological fluids such as blood, urine, CSF and breast milk [12]. They contain nucleic acids, proteins and lipids specific to their host cell, allowing for an astute reflection of the cell’s specific conditions [13,14,15,16]. In addition, they carry out various functions including the maintenance of cellular homeostasis [17], intercellular communication [18] and immunomodulation [19]. Tumour cells were also shown to transfer oncoproteins and RNAs to promote oncogenic transformation in neighbouring cells [20]. Several aspects of exosomes make them attractive candidates as biomarkers. Firstly, exosomes are considered critical indicators of cancers given their high specificity to the host cell and astute reflection of the biological state of its parent cell [14]. Measured changes can therefore be used to capture intra-tumour heterogeneity [14]. Secondly, exosomes are released in body fluids, which enable minimally invasive sampling [16]. Thirdly, they are highly stable in body fluids due to their lipid bilayer membrane which protects contents from degradation [16,21].

To date, there have been a number of studies exploring candidate biomarkers of HCC within exosomes, many of which rely on statistical inference to identify features correlated with HCC [22,23]. However, a limitation of these methods is that they are not designed to optimize predictive performance and involve assumptions about the data [24]. In addition, in the analysis of genetic data, there is often the issue of overfitting of models as the expression data typically have a small sample size and large number of features. Machine learning models overcome this through their ability to analyze large heterogenous datasets and predictive powers which set them apart from the traditional focus of statistical approaches [25]. Machine learning is a type of artificial intelligence that has emerged as a powerful discipline in medical research. It builds analytical models by analyzing existing data, and learns by observations with the primary purpose of making decisions on its own in the future. Models can be trained and automated to analyze multidimensional data for classification, clustering and predictive purposes [26,27]. Classification is a supervised learning approach in machine learning which is used to analyze a dataset provided and construct a model to divide data into a unique set of categories [28]. Among the classification techniques in machine learning, Supported Vector Machine (SVM) has been used as an effective tool in addressing binary classification problems in real world applications [29,30,31,32,33,34,35]. In SVM, the decision functions are determined directly from the dataset provided (training data) with the aim to maximize the separation (margin) between the decision borders in a highly dimensional space (feature space) [29].

As far as we are aware, only two studies have investigated the use of machine learning to identify or evaluate diagnostic and prognostic exosomal markers of HCC [36,37]. However, either the studies identify exosome-related genes from tumour tissue samples or machine learning was only used to evaluate three RNA detection panels for liver cancer.

**Figure 1:**
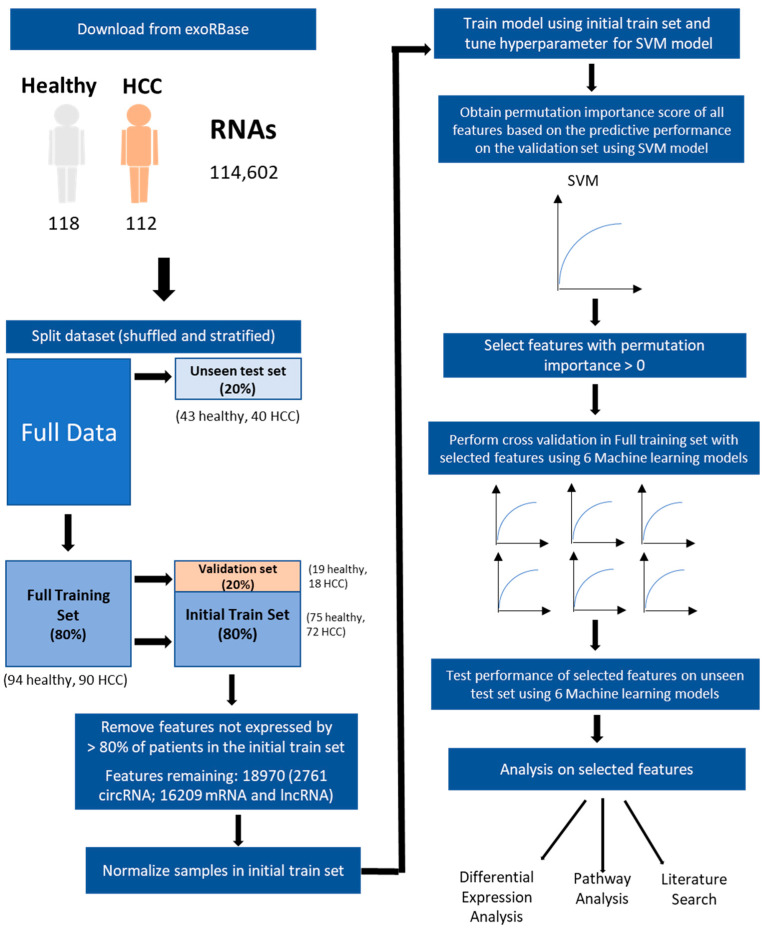
Overview of Model Building and Predictive Feature Selection Process of the Exosomes of HCC patients. The figure illustrates the process to build the predictive model and select relevant exosomal features. A total of 114,602 RNA expression profiles were obtained from 118 healthy individuals and 112 patients with hepatocellular carcinoma (HCC) in exoRBase. Data Splitting: The complete dataset was divided into an unseen test set (20%) and a full training set (80%). Further division of the training set created a validation set (20%) and an initial train set (80%). Data Preprocessing: Features that were not expressed in >80% of samples within the initial train set were removed, resulting in 18,970 remaining RNAs. Subsequently, the initial train set underwent normalization. Model Training: An SVM (Support Vector Machine) model was used to train the initial train set, and the hyperparameters were tuned for optimal performance. Feature Selection: Permutation importance scores were calculated for all features based on their predictive performance on the validation set. Features with permutation importance scores greater than 0 were considered as potential predictive features. Model Evaluation: Six different Machine Learning models were employed to assess the predictive capabilities of the potential predictors. Their predictive performance was evaluated on both the full training set and the unseen test set. Biological Significance Analysis: To evaluate the biological relevance of the potential predictors, additional analyses were conducted, including differential expression analysis, pathway analysis, and literature search.

In this study, we propose that machine learning can be used to identify biologically significant biomarkers based on exosomal RNA expression to predict HCC. Therefore, the aims of this study are first to identify the most predictive exosomal signatures of HCC from a model that integrates exosome mRNA, lncRNA and circRNAs, followed by evaluating their biological relevance to HCC. The strategy used is shown in Figure 1.

## 2. Materials and Methods

### 2.1. Exosomal RNA Expression Data

Exosome RNA (circRNA, mRNA and LncRNA) expression profiles from blood samples of HCC patients and healthy controls were downloaded from exoRBase 2.0 [38,39] (http://www.exorbase.org/ (accessed on 15 August 2022)) which contains RNA sequencing data of exosomal RNAs. The total dataset contains the expression of 114,602 RNAs consisting of 35,518 mRNAs/lncRNAs and 79,084 circRNAs from the blood samples of 112 HCC patients and 118 healthy controls.

### 2.2. Splitting and Processing of Data

The dataset was shuffled and split in 80:20 ratio for the train and unseen test set using the Scikit-learn module in Python (3.9.12). (Figure 1) The data were split in a stratified manner to retain the ratio between groups in each set.

### 2.3. Model Training and Feature Selection by Permutation Importance

The training set was further split 80–20 into a subset (hereon referred to as the initial train set) and a validation set, respectively. Features that were not expressed in more than 80% of patients in the initial train set were removed to reduce computational load and prevent inclusion of noise. The initial train set was scaled by sample to unit l2-norm using the Normalizer() function in Scikit-learn module [40], as this was reported to maximize accuracy and reduce fit time [41]. A Support Vector Machine (SVM) model was trained on the initial train set using GridSearchCV. The hyperparameters were further tuned to optimize its accuracy at predicting the validation set.

Due to the use of a non-linear SVM kernel, permutation importance was used to rank features. This method involves permutating data one feature at a time to calculate the importance of a feature based on the decrease in model score, which we defined as accuracy. As each permutation is random, this process was iterated three times before calculating the average permutation importance score for each feature. Features with an average permutation importance greater than 0 were selected for further evaluation.

### 2.4. Evaluating the Predictiveness of Selected Features

#### 2.4.1. Evaluation with Permutation Test

After feature selection, the selected features were evaluated using permutation test score from Scikit-learn module. In this test, the selected features were first extracted from the original training set, followed by training with 5-fold cross validation using SVM model. The significance of the performance of this trained model was then evaluated by comparing mean performance score of the original data and permutated datasets which have labels that were randomly shuffled 1000 times. Then, the empirical *p*-value between model performance on the original and the permutated set was calculated.

#### 2.4.2. Evaluation across 6 Different Models

Nine selected features were evaluated across six models in the full training and unseen test sets. Apart from SVM, the other models include random forest, multilayer perceptron (MLP), logistic regression, Gaussian naïve bayes and K-nearest neighbour. The hyperparameters of each model were tuned by GridSearchCV to maximize accuracy and evaluated by 5-fold cross-validation with the best estimator. Final assessment of the 6 models was based on prediction of the unseen test set; metrics include accuracy, ROC-AUC, specificity, sensitivity and F1 score.

### 2.5. Analysing Differential Gene Expression in Exosomal RNA Expression Data

Differential expression analysis was performed on exosomal RNA expression data. Fold change was calculated between HCC patients and healthy controls. Wilcoxon rank-sum test was performed on log2-normalized values to compare expression between the two groups. *p* values were adjusted using False Discovery Rate correction. The threshold for differential expression was set at absolute fold change >1.2 and adjusted *p* < 0.05.

### 2.6. Validation of Differential Expression and Predictive Performance of Potential Predictors in Tissues Samples

Tissue RNA sequencing files were downloaded from The Cancer Genome Atlas (TCGA) database (https://www.cancer.gov/tcga (accessed on 20 September 2022)). Transcript per million (TPM)-normalized data from 50 tumours matched with 50 adjacent non-tumour samples from 50 HCC patients were used for differential expression analysis. Fold change was calculated with the TPM values of each gene between matched tumour and adjacent non-tumour samples. Paired T test was performed on the log2-normalized values. *p* values were corrected for multiple testing using the Benjamin–Hochberg method. The threshold for differential expression was set at absolute fold change >1.2 and adjusted *p* < 0.05.

### 2.7. Pathway Enrichment Analysis

Over-representation analysis was performed on the selected predictive features using ConsensusPathDB (release 35) [42,43] to visualize their potential functions and pathways as defined by Reactome [44] and Kyoto Encyclopedia of Genes and Genomes (KEGG) [45].

### 2.8. Text Mining Analysis

To gain insight into the roles of the selected predictive features in HCC, Biopython [46] was used to search and return the PubMed IDs of articles that contained feature names or their alias and “HCC”, “LIHC” or “hepatocellular carcinoma” within the abstract. Features were annotated based on the reported association to HCC and whether any functional experiments were performed. Articles were excluded if features were not directly relevant to HCC and if features were only mentioned as housekeeping genes.

## 3. Results

### 3.1. Nine Exosomal RNA Signatures Selected by Machine Learning Approach Have Good Predictive Performance in Predicting HCC

The exosomal RNA expression data of 230 samples (118 healthy, 112 HCC) were first split into full training and unseen test set. The full training set consists of 184 samples (94 healthy; 90 HCC) while the unseen test set contains 46 samples (24 healthy; 22 HCC). The full training set was further split into initial train (75 healthy; 72 HCC) and validation set (19 healthy; 18 HCC) for feature selection (Figure 1).

Features with 0 expression in more than 80% of the samples were removed from the initial train set, which reduced features to 18,970. After fine-tuning the SVM model on the initial train set, the best parameters were found to be kernel = polynomial, cost = 10, gamma = scale. Permutation importance was then used to identify exosomal RNAs that are important in prediction. As a result, nine features had positive importance scores (Average permutation importance score > 0) and the best predictive performance with ROC-AUC of 0.89 in the validation set (Figure S1).

**Figure 2:**
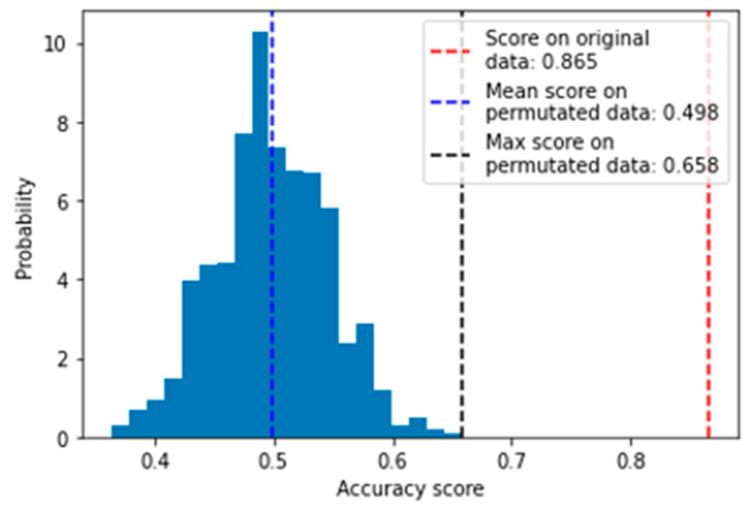
Distribution of accuracies obtained after 5-fold cross validation with SVM classifier on permutated data (*n* = 1000). The mean and maximum accuracy of the permutation test are indicated by the blue and black dotted lines, respectively. The accuracy obtained for cross validation on the non-permutated data is indicated by the red dotted line.

To evaluate the validity of the result and ensure that the good predictive performance is not due to random chance, the features were further evaluated with a permutation test using 5-fold cross validation of the SVM model on the full training set. The model with selected features predicted the full training set with an accuracy of 0.865, which was significantly greater than that for the permutated dataset (mean accuracy = 0.498, *p* = 9.99 × 10^−3^) (Figure 2). Therefore, accuracy of the SVM model was significantly better than random prediction.

**Figure 3:**
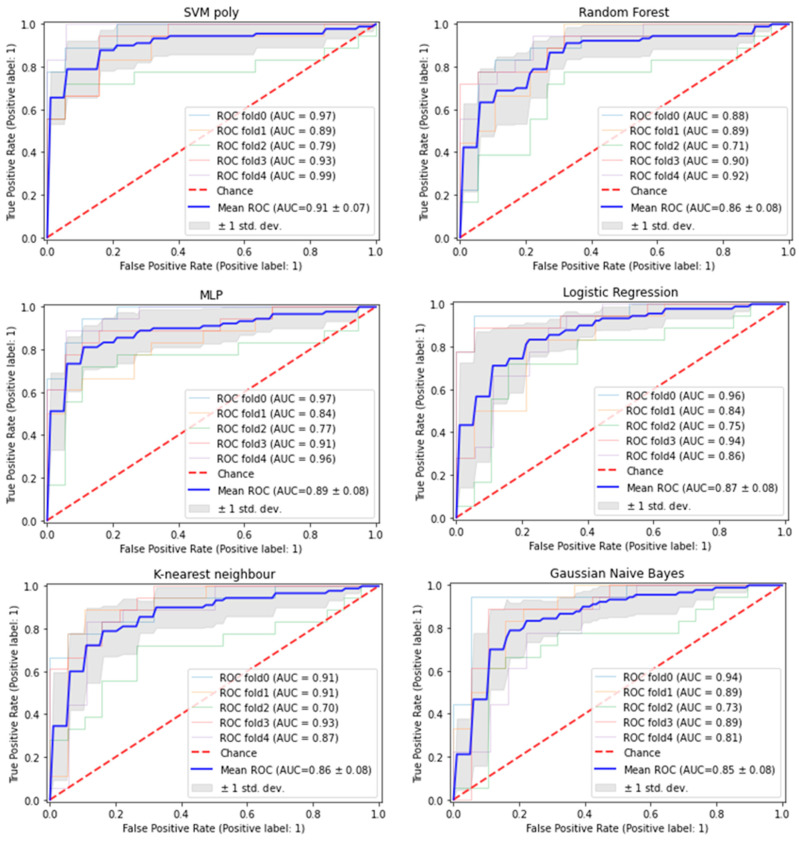
Predictive performance of nine exosomal features in training set across six ML models. The figure shows ROC curve graphs that represent performance of SVM (**top left**), Random Forest (**top right**), multilayer perceptron (MLP) (**middle left**), Logistic Regression (**middle right**), K-nearest neighbour (**bottom left**) and Gaussian naïve bayes (**bottom right**) models. The five solid lines in light pastel colours represent the ROC curves that were obtained from each fold (fold0–4) of the 5-fold cross-validation while the blue solid line represents the average ROC curve of the 5-fold cross-validation. The grey shade represents the standard deviation from the 5-fold cross-validation results. The red dotted line serves as a reference point which indicates that the model’s prediction is based on chance.

The nine features were also evaluated across five other machine learning (ML) models and all models achieved ROC-AUCs from 0.85–0.91 in the full training set (Figure 3).

Finally, the predictive performance of these nine features was evaluated using six ML models on the unseen test set. As a result, the nine features have good predictive performance with accuracies from 0.76–0.85 and ROC-AUC from 0.79–0.88 in all six ML models (Table 1).

### 3.2. The Nine ML Selected Exosomal RNA Signatures Performs Better than Top Nine Differentially Expressed RNAs

Seven of nine exosomal RNA signatures selected by the ML method are mRNAs (Table 2) while two are circRNAs which are derived from the exons of their parent genes (Table 3).

Of these, MTRNR2L8, S100A11, S100A9 and exo_circ_79050 were differentially expressed between HCC patients and healthy individuals using an absolute fold change threshold >1.2 and adjusted *p* < 0.05 (Red box in Table S1). Given that only 4/9 potential predictors identified by ML feature selection method are differentially expressed, an additional analysis was conducted to evaluate the predictive performance of the top nine differentially expressed exosomal RNAs with the greatest absolute fold change and adjusted *p* value < 0.05 (Table S2) across the same six ML models. As a result, the predictive performance in the unseen test set decreased across all six models as the accuracies are less than 0.70 while the ROC-AUC values are less than 0.80 (Table S3) except for the Random Forest model which had accuracy of 0.78 and ROC-AUC of 0.85. These results demonstrate the robustness in prediction of HCC by the potential predictors selected by ML feature selection method.

### 3.3. Majority of the Exosomal RNA Signatures Are also Differentially Expressed in Tumour Tissues as Compared to Adjacent Non-Tumourous Tissues

As exosomal RNAs were shown to be secreted by tumour cells and to contain molecular information that reflect the biological state of their parent cells [14,20], we investigated if the seven exosomal mRNAs from the nine ML selected features and parental genes of the two ML selected exosomal circRNAs are differentially expressed in tumour versus adjacent non-tumour tissues from TCGA dataset. Using fold change >1.2 and adjusted *p* < 0.05, six mRNAs and both parental genes of circRNAs are differentially expressed in tumour versus non-tumour tissues (Red box in Table S4). This suggests that the 8/9 exosomal mRNAs that are mainly detected in blood exosomes could also reflect the deregulated expression in the tumour tissues of the patients.

### 3.4. ML-Selected Exosomal RNA Signatures Are Mainly Implicated in Immune Pathways and Majority Are Previously Reported to Be Associated with HCC

**Figure 4:**
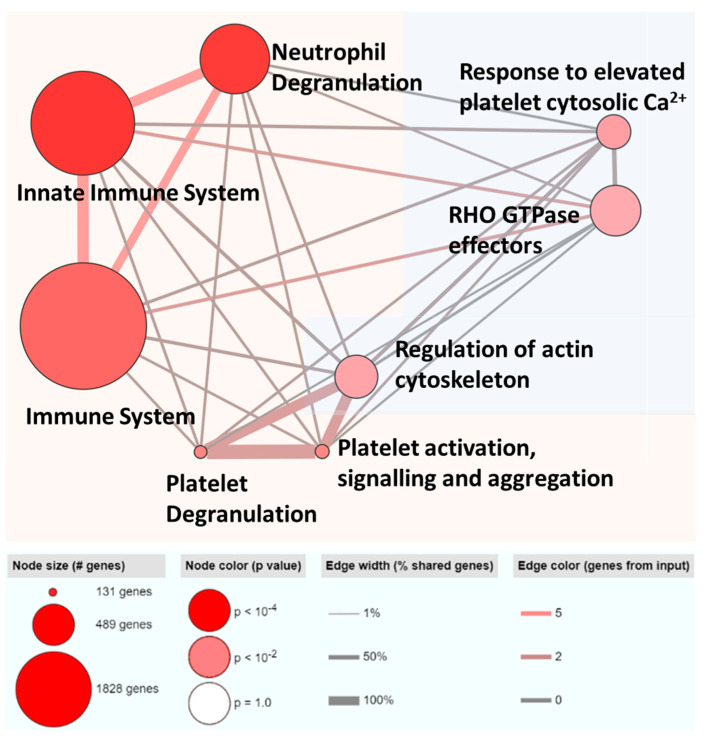
Over-representation analysis of nine exosomal RNAs in ConsensusPathDB. Nodes grouped within the red shaded box are immune related. Nodes grouped within the blue shaded box are cytoskeleton related.

To obtain insights on the biological significance of the exosomal RNA signatures, seven exosomal mRNAs and the parent genes of the two exosomal circRNAs were mapped on ConsensusPathDB using over-representation analysis (Figure 4). Most pathways identified were immune-related while the other pathways were involved in regulation of the cytoskeleton.

The nine exosomal RNA signatures were also searched in Pubmed using Biopython in order to gain insight into their relevance in HCC in the previous literature. Of nine features, seven have been reported in the literature to be associated with HCC (Table S5). On the other hand, MTRNR2L8 and exo_circ_79050 were not reported to be associated with HCC previously, suggesting that these two features could be novel mRNA or circRNA in HCC.

## 4. Discussion

The new frontier in biomarker research is the development of panels instead of a single marker for the detection of cancer [47]. This is supported by evidence on how the use of a panel may improve accuracy and predictive performance [48]. Hence, in this study, we employed a machine learning approach to identify a panel of nine exosomal RNA signatures which included seven exosomal mRNAs and two exosomal circRNAs that distinguished HCC patients from healthy controls with good predictive performance. We showed that the best machine learning model with nine exosomal RNAs signatures distinguished HCC patients from an unseen test with the highest accuracy of 85% and ROC-AUC of 0.88 (Red box in Table 1). The performance of our model was significantly more accurate at predicting HCC as shown in the permutation test (Figure 2) and therefore this result is not by chance.

Conventionally, biomarkers have been selected based on differential expression between cancer and non-cancer samples. However, only four of the nine potential predictors identified by ML feature selection methods are differentially expressed in exosomes between HCC patients as compared to healthy controls. When we evaluated the predictive performance of six ML models trained on nine exosome features with the highest absolute fold change, the prediction accuracy on the unseen test set was lower as compared to the predictive accuracy using the nine ML-selected exosomal RNA signatures. This is likely because analysing differential expressed RNAs independently provides limited biological insight. For example, it is known that slight changes in the expression of hub genes can critically affect important pathways in various diseases and therefore may be more predictive [49]. However, these genes may be filtered out in differential expression analysis if their effect size is too small [50].

Notably, the majority of the exosomal RNA signatures (eight exosomal mRNAs including parental genes of circRNAs) were differentially expressed in tumour tissues as compared to adjacent non-tumour tissues in TCGA dataset. This result suggests that the potential predictors could be oncogenes or tumour suppressors that are secreted from tissue samples to exosomes and may therefore also act as potential biomarkers for HCC. Nonetheless, future studies are required to validate their potential oncogenic or tumour-suppressing effects.

It is interesting to note that, although the potential predictors were identified by a machine learning approach, the exosomal RNA signatures are biologically relevant, as shown in pathway analysis and the text mining approach. Enrichment analysis indicated that seven exosomal mRNAs and the parent genes of the remaining two exosomal circRNAs converged on immune pathways. This is consistent with the understanding that exosomes can regulate immune components [51] while the immune contexture of HCC has been shown to be important for predicting clinical outcomes [52].

Of the 9 features, 7/9 have also been associated with HCC in the past literature (Table S5). MTRNR2L8, which was identified as the most important feature in our predictive model (Table 2), has not yet been implicated in HCC. However, it has been reported to be significantly downregulated in breast cancer and is likely to interact with lncRNA NEAT1, which has been found to drive the progression of various cancers including colorectal, breast and gastric cancer [53]. Therefore, future study can be conducted to further investigate the potential role of MTRNR2L8 in HCC.

On the other hand, exo_circ_79050 was identified as the most differentially expressed RNA in the exosome (Table S2 and Table 3). This circRNA is derived from the Y-linked pseudogene TXLNGY, and, since males are at a greater risk of developing HCC, it is likely that its differential expression is in part due to a greater proportion of males among HCC patients compared to healthy controls. Although we did not find any studies to suggest its involvement nor the involvement of its parent gene in HCC, the parental gene TXLNGY has been reported in other male-dominated cancers. One study found that downregulation of TXLNGY and Y disruption in the tumour are associated with poor prognosis in male-dominant cancers such as lung cancer [54]. Therefore, a follow up of this study would be to determine the predictive value of exo_circ_79050 for diagnosing HCC in a male-only cohort. Additionally, future study can be carried out to evaluate if removing this feature would improve accuracy in HCC prediction among females. Taken together, these findings suggest that predictive exosomal RNA signatures are biologically relevant to HCC or other cancers.

Future studies are required to validate the predictive performance of the nine exosomal RNA signatures in larger and independent cohorts. To further improve our prediction model, it is worthwhile exploring whether the exosomal RNA signatures could be further reduced by incorporating clinical information in the machine learning models in the future.

## 5. Conclusions

Overall, this study shows that exosomal RNA signatures identified by a machine learning approach with good predictive performance could act as potential biomarkers of HCC. Moreover, these features are not just artefacts of a single model but are likely to have biological significance.

## 6. Patents

We are in the process of obtaining a patent for this study. The identity of the genes and circRNAs will be revealed once the IP is obtained.

## Figures and Tables

**Table 1:** Performance of nine exosomal RNAs in predicting HCC vs. healthy patients in unseen test set.

Model	SVM	MLP	Random Forest	Logistic Regression	K-Nearest Neighbour	Gaussian Naïve Bayes
Accuracy	0.761	0.761	0.848	0.783	0.848	0.761
Precision	0.790	0.739	0.941	0.833	0.941	0.867
Sensitivity	0.682	0.773	0.727	0.682	0.727	0.591
Specificity	0.833	0.750	0.958	0.875	0.958	0.917
FPR	0.167	0.250	0.042	0.125	0.0417	0.083
F1-Score	0.732	0.756	0.821	0.750	0.821	0.703
AUC	0.840	0.850	0.870	0.810	0.880	0.790

Red border: ML model with the best performance.

**Table 2:** Annotation of selected mRNA features.

Exosome RNA	Gene Ensemble ID	Name	Mean Importance	Importance Rank
MTRNR2L8	ENSG00000255823.4	MT-RNR2 Like 8	0.162	1
FTL	ENSG00000087086.14	Ferritin Light Chain	0.090	2
PPBP	ENSG00000163736.3	Pro-Platelet Basic Protein	0.027	4
TMSB4X	ENSG00000205542.10	Thymosin Beta 4 X-Linked	0.018	5
S100A11	ENSG00000163191.5	S100 Calcium Binding Protein A11	0.018	6
S100A9	ENSG00000163220.10	S100 Calcium Binding Protein A9	0.009	7
ACTB	ENSG00000075624.14	Actin Beta	0.009	8

**Table 3:** Annotation of selected circRNA features.

exoRBase circID	circBase ID	Genomic Position	Strand	Parent Gene Symbol	Parent Gene Type	Mean Importance	Importance Rank
exo_circ_22106	hsa_circ_000072	chr16:85633914-85634132 (exon)	+	GSE1	protein coding	0.036	3
exo_circ_79050	hsa_circ_0009024	chrY:19587210-19587507 (exon)	+	TXLNGY	pseudogene	3.70 × 10^−17^	9

## Data Availability

The script and codes that support the findings of this study are available on https://github.com/LCFGChipmunt/mlexosomalrna.

## Supplementary Materials

The following supporting information can be downloaded at: https://www.mdpi.com/article/10.3390/cancers15143749/s1, Figure S1. Predictive performance of nine machine learning selected exosomal RNAs in validation set; Table S1. Differential expression of nine machine learning selected exosomal RNAs signatures between HCC patients and healthy individuals; Table S2. Top nine exosomal RNAs that are significantly differentially expressed with the highest absolute fold change between HCC patients and healthy individuals; Table S3. Predictive performance of top nine significantly differentially expressed exosomal RNAs in predicting HCC patients vs. healthy individuals in unseen test set; Table S4. Differential expression of the nine exosomal RNA signatures between tumour and adjacent non-tumour samples in TCGA data; Table S5. Existing literature of nine exosomal RNA signatures in HCC.
