# Supplementary material for: Machine Learning Identifies a Signature of Nine Exosomal RNAs That Predicts Hepatocellular Carcinoma
Source: Cancers (Basel). 2023 Jul 24;15(14):3749. doi: 10.3390/cancers15143749 (PMC10377993; doi:10.3390/cancers15143749)
Supplement: Supplementary file 1 [file cancers-15-03749-s001.zip › cancers-2481756-supplementary.pdf]

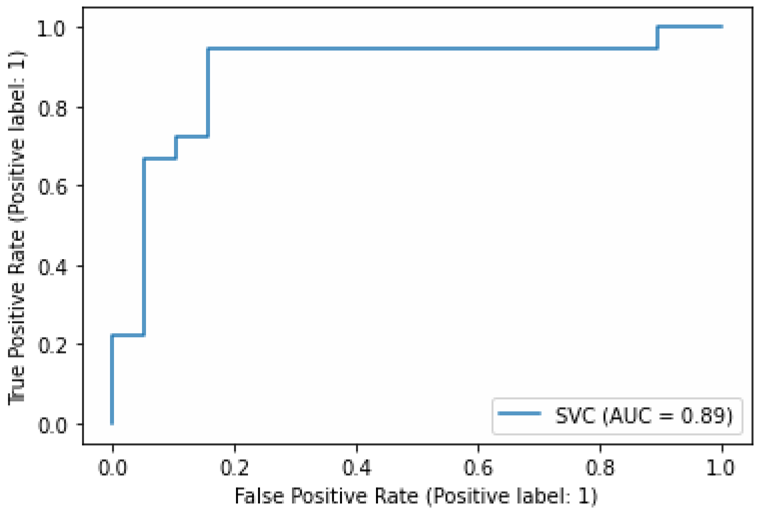

Figure S1. Predictive performance of nine machine learning selected exosomal RNAs in validation set.

Table S1. Differential expression of nine machine learning selected exosomal RNAs signatures between HCC patients and healthy individuals.

**Exosomal RNAs**	**Mean Expression Log2(TPM+1)**		**Wilcoxon Rank Sum test**		
	**HCC**	**Healthy**	**Fold Change**	***P* value**	**Adjusted *P***
MTRNRL8	13.59	12.57	2.02	1.81E-06	1.76E-04
FTL	13.88	13.83	1.04	1.57E-01	3.50E-01
PPBP	13.8	13.94	-1.1	6.61E-01	8.10E-01
TMSB4X	13.48	13.59	-1.08	1.96E-01	4.01E-01
S100A11	11.87	11.29	1.49	1.49E-06	1.50E-04
S100A9	12.21	11.53	1.6	3.78E-08	7.00E-06
ACTB	14.19	14.11	1.06	2.00E-01	4.04E-01
GSE1 (parental gene of exo_circ_22106)	6.73	6.95	-1.17	7.21E-04	1.17E-02
TXLNGY (parental gene of exo_circ_79050)	N/A	N/A	N/A	N/A	N/A
exo_circ_22106	12.64	12.79	-1.11	7.85E-02	2.30E-01
exo_circ_79050	8.05	4.67	10.46	2.05E-08	4.00E-06

N/A: Data is not available; Red box: Exosomal RNAs that passed the criteria of absolute fold change threshold of 1.2 and adjusted p<0.05.

Table S2. Top nine exosomal RNAs that are significantly differentially expressed with the highest absolute fold change between HCC patients and healthy individuals.

Exosomal RNAs	Mean Expression Log2(TPM+1)		Wilcoxon Rank Sum test		
	HCC	Healthy	Fold Change	*P* value	Adjusted *P*
exo_circ_79050	8.05	4.67	10.46	2.05E-08	4.02E-06
exo_circ_79066	6.98	3.93	8.31	1.19E-06	1.26E-04
exo_circ_71478	6.81	3.96	7.18	6.49E-06	4.53E-04
exo_circ_11335	6.42	3.6	7.08	1.95E-06	1.85E-04
exo_circ_68977	6.77	3.98	6.92	5.59E-06	4.13E-04
exo_circ_38752	6.61	3.87	6.69	7.01E-05	2.45E-03
ORM1	3.78	1.07	6.52	2.11E-18	6.68E-15
exo_circ_19955	6.81	4.27	5.8	4.23E-05	1.71E-03
exo_circ_71780	3.75	1.23	5.74	4.31E-07	5.26E-05

Table S3. Predictive performance of top nine significantly differentially expressed exosomal RNAs in Predicting HCC patients vs. healthy individuals in unseen test set.

	SVM	MLP	Random Forest	Logistic Regression	K-nearest neighbour	Gaussian Naïve Bayes
Accuracy	0.587	0.652	0.783	0.609	0.696	0.587
Precision	0.556	0.600	0.750	0.567	0.654	0.548
Recall	0.618	0.818	0.818	0.773	0.773	0.773
Specificity	0.500	0.500	0.750	0.458	0.625	0.417
FPR	0.500	0.500	0.250	0.542	0.375	0.583
F1-Score	0.612	0.692	0.783	0.654	0.708	0.642
AUC	0.740	0.710	0.850	0.750	0.730	0.620

Table S4. Differential expression of the nine exosomal RNA signatures between tumour and adjacent non-tumour samples in TCGA data.

Exosomal RNAs	Mean Expression Log2(TPM+1)		Paired T-test		
	HCC Tumour	Adjacent non-tumour	Fold Change	*P* value	Adjusted *P*
MTRNRL8	1.54	1.84	-1.17	3.99E-03	7.31E-03
FTL	31919.76	15835.75	1.4	1.11E-02	1.82E-02
PPBP	0.37	0.93	-1.42	1.31E-05	4.06E-05
TMSB4X	731.6	502.14	1.24	8.44E-02	1.11E-01
S100A11	351.79	98.25	1.91	1.09E-03	2.27E-03
S100A9	59.81	59.53	-1.78	2.73E-03	5.19E-03
ACTB	1710.52	1041.64	1.58	1.49E-07	6.69E-07
GSE1 (parental gene of exo_circ_22106)	8.49	5.73	1.34	9.42E-04	1.99E-03
TXLNGY (parental gene of exo_circ_79050)	1.67	0.93	1.2	0.0113	0.0186
exo_circ_22106	N/A	N/A	N/A	N/A	N/A
exo_circ_79050	N/A	N/A	N/A	N/A	N/A

N/A: Data is not available.

Table S5. Existing literature of nine exosomal RNA signatures in HCC.

Features	Pubmed ID	Association with HCC	Validated by experiment	Observation	Citation
TMSB4X	24299315	Y	Y	Upregulated in HCC	Yang, L., Ji, J., Chen, Z., Wang, H. and Li, J. (2014). Transcriptome profiling of malignant transformed rat hepatic stem-like cells by aflatoxin B1. *Neoplasma*, *61*(2), 193–204. https://doi.org/10.4149/neo_2014_025
	29568350	Y	Y	Promotes angiogenesis	Lee, H. Y., Chen, C. K., Ho, C. M., Lee, S. S., Chang, C. Y., Chen, K. J. and Jou, Y. S. (2018). EIF3C-enhanced exosome secretion promotes angiogenesis and tumorigenesis of human hepatocellular carcinoma. *Oncotarget*, *9*(17), 13193–13205. https://doi.org/10.18632/oncotarget.24149
	31703598	Y	Y	Promotes metastasis	Dong, Y., Zheng, Q., Wang, Z., Lin, X., You, Y., Wu, S., Wang, Y., Hu, C., Xie, X., Chen, J., Gao, D., Zhao, Y., Wu, W., Liu, Y., Ren, Z., Chen, R. and Cui, J. (2019). Higher matrix stiffness as an independent initiator triggers epithelial-mesenchymal transition and facilitates HCC metastasis. *Journal of hematology & oncology*, *12*(1), 112. https://doi.org/10.1186/s13045-019-0795-5
S100A11	31919231	Y	Y	Promotes inflammation	Sobolewski, C., Abegg, D., Berthou, F., Dolicka, D., Calo, N., Sempoux, C., Fournier, M., Maeder, C., Ay, A. S., Clavien, P. A., Humar, B., Dufour, J. F., Adibekian, A. and Foti, M. (2020). S100A11/ANXA2 belongs to a tumour suppressor/oncogene network deregulated early with steatosis and involved in inflammation and hepatocellular carcinoma development. Gut, 69(10), 1841–1854. https://doi.org/10.1136/gutjnl-2019-319019
	33815482	Y	N	Upregulation associated with poor survival Immune regulation	Zheng, S., Liu, L., Xue, T., Jing, C., Xu, X., Wu, Y., Wang, M., Xie, X. and Zhang, B. (2021). Comprehensive Analysis of the Prognosis and Correlations With Immune Infiltration of S100 Protein Family Members in Hepatocellular Carcinoma. *Frontiers in genetics*, *12*, 648156. https://doi.org/10.3389/fgene.2021.648156
	33546025	Y	N	Upregulation associated with poor survival	Zhang, C., Yao, R., Chen, J., Zou, Q. and Zeng, L. (2021). S100 family members: potential therapeutic target in patients with hepatocellular carcinoma: A STROBE study. *Medicine*, *100*(3), e24135. https://doi.org/10.1097/MD.0000000000024135

S100A9	33815482	Y	N	Downregulated in tumour	Zheng, S., Liu, L., Xue, T., Jing, C., Xu, X., Wu, Y., Wang, M., Xie, X. and Zhang, B. (2021). Comprehensive Analysis of the Prognosis and Correlations With Immune Infiltration of S100 Protein Family Members in Hepatocellular Carcinoma. *Frontiers in genetics*, *12*, 648156. https://doi.org/10.3389/fgene.2021.648156
	33546025	Y	N	Upregulation associated with poor survival	Zhang, C., Yao, R., Chen, J., Zou, Q. and Zeng, L. (2021). S100 family members: potential therapeutic target in patients with hepatocellular carcinoma: A STROBE study. *Medicine*, *100*(3), e24135. https://doi.org/10.1097/MD.0000000000024135
	35397536	Y	Y	Upregulation associated with poor survival Immune regulation	Wang, Y., Yang, Y., Zhao, Z., Sun, H., Luo, D., Huttad, L., Zhang, B. and Han, B. (2022). A new nomogram model for prognosis of hepatocellular carcinoma based on novel gene signature that regulates cross-talk between immune and tumor cells. *BMC cancer*, *22*(1), 379. https://doi.org/10.1186/s12885-022-09465-9
	35560794	Y	N	Immune regulation	Chen, J., Wang, H., Zhou, L., Liu, Z., Chen, H. and Tan, X. (2022). A necroptosis-related gene signature for predicting prognosis, immune landscape and drug sensitivity in hepatocellular carcinoma. *Cancer medicine*, 10.1002/cam4.4812. Advance online publication. https://doi.org/10.1002/cam4.4812
	35693827	Y	N	Prognostic marker	Wang, T., Dai, L., Shen, S., Yang, Y., Yang, M., Yang, X., Qiu, Y., & Wang, W. (2022). Comprehensive Molecular Analyses of a Macrophage-Related Gene Signature With Regard to Prognosis, Immune Features and Biomarkers for Immunotherapy in Hepatocellular Carcinoma Based on WGCNA and the LASSO Algorithm. *Frontiers in immunology*, *13*, 843408. https://doi.org/10.3389/fimmu.2022.843408
	35992798	Y	N	Prognostic marker Immune regulation	Yuan, J., Wang, Y., Wang, X., Zhang, W., Ding, R., Yue, S. and Li, X. (2022). Construction and experimental verification of user-friendly molecular subtypes mediated by immune-associated genes in hepatocellular carcinoma. *Frontiers in oncology*, *12*, 924059. https://doi.org/10.3389/fonc.2022.924059

	36041055	Y	Y	Upregulation associated with recurrence Promotes growth and EMT	Zhong, C., Niu, Y., Liu, W., Yuan, Y., Li, K., Shi, Y., Qiu, Z., Li, K., Lin, Z., Huang, Z., Zuo, D., Yang, Z., Liao, Y., Zhang, Y., Wang, C., Qiu, J., He, W., Yuan, Y. and Li, B. (2022). S100A9 Derived from Chemoembolization-Induced Hypoxia Governs Mitochondrial Function in Hepatocellular Carcinoma Progression. *Advanced science (Weinheim, Baden-Wurttemberg, Germany)*, e2202206. Advance online publication. https://doi.org/10.1002/advs.202202206
	36032071	Y	N	Prognostic marker Immune regulation	Guo, C., Tang, Y., Yang, Z., Li, G. and Zhang, Y. (2022). Hallmark-guided subtypes of hepatocellular carcinoma for the identification of immune-related gene classifiers in the prediction of prognosis, treatment efficacy and drug candidates. *Frontiers in immunology*, *13*, 958161. https://doi.org/10.3389/fimmu.2022.958161
PPBP	31346321	Y	N	Diagnostic marker	Ding, B., Lou, W., Liu, J., Li, R., Chen, J. and Fan, W. (2019). In silico analysis excavates potential biomarkers by constructing miRNA-mRNA networks between non-cirrhotic HCC and cirrhotic HCC. *Cancer cell international*, *19*, 186. https://doi.org/10.1186/s12935-019-0901-3
	31432149	Y	N	Prognostic marker	Wang, X., Zhou, X., Liu, J., Liu, Z., Zhang, L., Gong, Y., Huang, J., Yu, L., Wang, Q., Yang, C., Liao, X., Yu, T., Han, C., Zhu, G., Ye, X. and Peng, T. (2019). Genome-wide investigation of the clinical implications and molecular mechanism of long noncoding RNA LINC00668 and protein-coding genes in hepatocellular carcinoma. *International journal of oncology*, *55*(4), 860–878. https://doi.org/10.3892/ijo.2019.4858
FTL	32577166	Y	N	Diagnostic marker	Sayeed, A., Dalvano, B. E., Kaplan, D. E., Viswanathan, U., Kulp, J., Janneh, A. H., Hwang, L. Y., Ertel, A., Doria, C. and Block, T. (2020). Profiling the circulating mRNA transcriptome in human liver disease. *Oncotarget*, *11*(23), 2216–2232. https://doi.org/10.18632/oncotarget.27617
	34790741	Y	Y	Promotes growth	He, Y., Fang, D., Liang, T., Pang, H., Nong, Y., Tang, L., Yang, Z., Lu, C., Han, X., Zhao, S., Mo, S., Meng, Y., Han, C. and Peng, T. (2021). Atractylodin may induce ferroptosis of human hepatocellular carcinoma cells. *Annals of translational medicine*, *9*(20), 1535. https://doi.org/10.21037/atm-21-4386

	33864445	Y	N	Upregulation associated with poor survival	Hu, Z. W., Chen, L., Ma, R. Q., Wei, F. Q., Wen, Y. H., Zeng, X. L., Sun, W. and Wen, W. P. (2021). Comprehensive analysis of ferritin subunits expression and positive correlations with tumor-associated macrophages and T regulatory cells infiltration in most solid tumors. *Aging*, *13*(8), 11491–11506. https://doi.org/10.18632/aging.202841
	35378780	N	N	Biomarker of Liver Cancer	Ren, P., Wang, K., Ma, J., Cao, X., Zhao, J., Zhao, C., Guo, Y. and Ye, H. (2022). Autoantibody Against Ferritin Light Chain is a Serum Biomarker for the Detection of Liver Cirrhosis but Not Liver Cancer. *Journal of hepatocellular carcinoma*, *9*, 221–232. https://doi.org/10.2147/JHC.S352057
	35651950	Y	Y	Promotes growth. Prognostic marker Immune regulation	Ke, S., Wang, C., Su, Z., Lin, S. and Wu, G. (2022). Integrated Analysis Reveals Critical Ferroptosis Regulators and FTL Contribute to Cancer Progression in Hepatocellular Carcinoma. *Frontiers in genetics*, *13*, 897683. https://doi.org/10.3389/fgene.2022.897683
ACTB	31846694	Y	Y	Promotes metastasis	Li, Y., Ma, H., Shi, C., Feng, F. and Yang, L. (2020). Mutant ACTB mRNA 3'-UTR promotes hepatocellular carcinoma development by regulating miR-1 and miR-29a. *Cellular signalling*, *67*, 109479. https://doi.org/10.1016/j.cellsig.2019.109479
exo_circ_22106/ circGSE1	35396771	Y	Y	Immune regulation	Huang, M., Huang, X. and Huang, N. (2022). Exosomal circGSE1 promotes immune escape of hepatocellular carcinoma by inducing the expansion of regulatory T cells. *Cancer science*, *113*(6), 1968–1983. https://doi.org/10.1111/cas.15365

Y: Yes; N: No.
